# Simplified sequence-based method for ATP-binding prediction using contextual local evolutionary conservation

**DOI:** 10.1186/1748-7188-9-7

**Published:** 2014-03-11

**Authors:** Chun Fang, Tamotsu Noguchi, Hayato Yamana

**Affiliations:** 1 Department of Computer Science, Engineering of Waseda University, Tokyo, Japan; 2 Computational Biology Research Center (CBRC), Tokyo, Japan; 3 Meiji Pharmaceutical University, Tokyo, Japan

**Keywords:** ATP-binding site, Sequence-based, Local evolutionary conservation

## Abstract

**Background:**

Identifying ligand-binding sites is a key step to annotate the protein functions and to find applications in drug design. Now, many sequence-based methods adopted various predicted results from other classifiers, such as predicted secondary structure, predicted solvent accessibility and predicted disorder probabilities, to combine with position-specific scoring matrix (PSSM) as input for binding sites prediction. These predicted features not only easily result in high-dimensional feature space, but also greatly increased the complexity of algorithms. Moreover, the performances of these predictors are also largely influenced by the other classifiers.

**Results:**

In order to verify that conservation is the most powerful attribute in identifying ligand-binding sites, and to show the importance of revising PSSM to match the detailed conservation pattern of functional site in prediction, we have analyzed the Adenosine-5'-triphosphate (ATP) ligand as an example, and proposed a simple method for ATP-binding sites prediction, named as CLCLpred (Contextual Local evolutionary Conservation-based method for Ligand-binding prediction). Our method employed no predicted results from other classifiers as input; all used features were extracted from PSSM only. We tested our method on 2 separate data sets. Experimental results showed that, comparing with other 9 existing methods on the same data sets, our method achieved the best performance.

**Conclusions:**

This study demonstrates that: 1) exploiting the signal from the detailed conservation pattern of residues will largely facilitate the prediction of protein functional sites; and 2) the local evolutionary conservation enables accurate prediction of ATP-binding sites directly from protein sequence.

## Background

Identification of ligand-protein binding sites is a key step to annotate the protein functions and find applications in drug design. Since physical experimental methods are expensive and time consuming, computational methods are indispensable for guiding the physical experimental analysis.

So far, several computational methods have been proposed for identifying protein functional sites [1-17]. These methods can be categorized into three groups: 1) approaches that focus on molecular docking with known protein structures [1-5]; 2) methods that predict putative interacting sites based on protein sequences [6-17]; 3) methods that identify interacting sites based on the hybrid features of protein structure and sequences [15]. Due to the structures of most proteins are not available, the structure-based methods cannot be generally used. Here, we focus on the sequence-based methods of ligand-binding sites prediction. The inputs of previous sequence-based methods also can be categorized into three groups: (i) direct output of PSSM [6-10]; (ii) combination of PSSM with other sequence features, including amino acid distance and physicochemical prosperity [11] ; (iii) combination of PSSM with other predicted structural information, such as predicted secondary structure [12,16,17], predicted solvent accessibility [12,16,17], predicted disorder probabilities [16,17], predicted dihedral angles [16] and predicted B-factors. Of course, analyzing the various features of the binding partner is important for understanding the ligand-binding behavior. However, incorporating many other features, especially some predicted feature as input for binding sites prediction has some potential shortcomings: 1) multi-features easily result in high-dimensional feature space. In machine learning, if the training samples are limited, high-dimensional feature space easily leads to over fitting to noise data and then cause a degradation in performance; 2) incorporating predicted results from other predictors greatly increases the complexity of the algorithm. Many studies even provided no Web services because of the complexity in their design; 3) the performance of the predictors which employed other predicted results are also largely affected by other classifiers. Thus, more simple and high efficient method for identifying interacting residues is indispensable.

Evolutionary information included in PSSM has been considered to be the most effective feature for functional site predication. Nearly all the sequence-based methods adopted PSSM as an input feature for prediction. Raghava’s group has used the standard PSSMs for various functional sites predication [7-10]. John A. C. et al. [13] also found that conservation features was highly predictive in identifying ligand-binding sites and catalytic sites compared to identify other functional site. Ke Chen et al. [18] proved that the exclusion of PSSM profile leads to a larger decrease in prediction performance than the exclusion of other input features, which suggests that the evolutionary information plays a key role in determination of the nucleotide-binding residues.

Here, we developed a novel sequence-based predictor for ATP-binding sites prediction. In our approach, only the high local evolutionary conservation scores in the PSSMs are considered as input. We employed no other predicted results as input features. Our method is based on the assumptions that: 1) the most effective features for predicting functional sites are embedded in the sequence itself; 2) the local evolutionary conservation is distinct enough to enable an accurate prediction of ATP-binding sites directly from amino acid sequence, without requiring any additional predicted structural information. In order to assess the predictive quality, we compared our method with 9 other existing methods which adopted various predicted structural features. The Support Vector Machine (SVM) was adopted to build the classifiers.

## Methods

### Benchmark datasets

We took ATP as an example of ligands. For facilitating comparison, we collected 2 data sets from the existing papers [10,16,17].

Dataset 1: It is extracted from the reference [10], which was also used in the reference [17]. This data set includes 168 ATP-binding protein chains, that contain 3,104 ATP-interacting residues (AIRs) and 59,226 non-ATP interacting residues (non-AIRs), named as ATP168. It is available at http://www.imtech.res.in/raghava/atpint/.

Dataset 2: It is extracted from the reference [16], which was also used in the reference [17]. This data set includes 227 ATP-binding protein chains that contain 3,393 AIRs and 80,409 non-AIRs, named as ATP227. It is available at http://biomine.ece.ualberta.ca/ATPsite/.

### Continuous binding residues analysis

**Figure 1:**
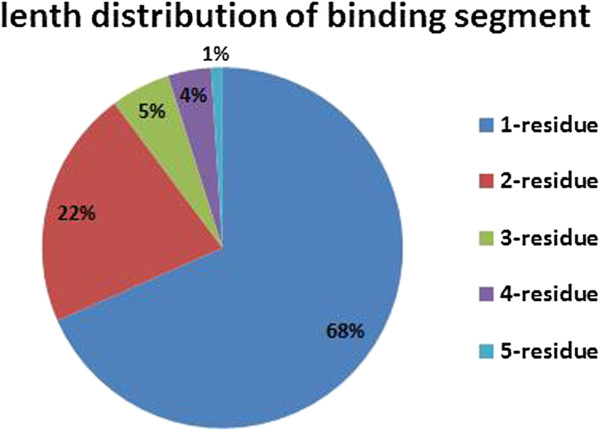
Statistics of continuous ATP-binding residues.

In order to conform whether ATP-binding sites are clustered closely together in sequence, the 168 ATP-binding protein chains in Dataset 1 were analyzed as example. As shown in Figure 1, 68% of binding-sites appear alone, 22% of them appear in two consecutive residues, and the others appear continuously with lengths distribution between 3 to 5 residues. This result suggests that, the ATP-binding sites have certain independence, and meanwhile are affected by their flanking regions to some extent.

### Composition and physicochemical properties analysis

We next analyzed the composition and physicochemical properties of the ATP-binding proteins. Sequences were divided into 3 regions: the ATP-interacting residues (AIRs), regions flanking the AIRs, and general non-ATP interacting regions (non-AIRs). Regions flanking on either side of the interacting sites with 15-residue long were analyzed.

**Figure 2:**
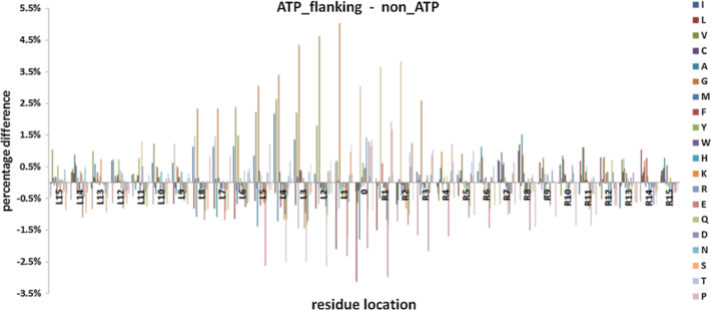
**Differences with respect to amino acids composition between AIRs and non-AIRs.** The values at position 0 on the horizontal axis mean the values for AIRs, L means the left flanking side and R means the right flanking side.

Difference between the flanking regions and general non-AIRs regions (percentage of flanking - percentage of non_AIRs) with respect to 20 amino acids composition is shown in Figure 2. The Pearson product-moment correlation coefficients between the flanking length and the composition difference for each amino acid were also calculated (Table 1 for the left flanking side and Table 2 for the right flanking side). For both the flanking regions, the correlation coefficients related to Leu, Gly, Glu, Gln, Ser, and Thr have absolute value > 0.69 at the *p*-value <0.001. It illustrates that, composition of these amino acids in the flanking regions and in the general non-binding regions is significantly different.

**Table 1:** Correlation coefficients between the left flanking length and composition difference (left ATP_flanking – non_ATP) for each amino acid

	**I**	**L**	**V**	**C**	**A**	**G**	**M**	**F**	**Y**	**W**
Correlation coefficient	0.436261	**-**0.69187	0.205958	-0.44066	5.56E-05	0.89866	-0.4487	-0.03841	-0.37415	0.182664
p-value	9.12E-02	2.98E-03	0.4441	0.08756	-0.83563	2.24E-06	8.13E-02	8.88E-01	1.53E-01	4.98E-01
	H	K	R	E	Q	D	N	**S**	T	P
Correlation coefficient	-0.19525	-0.449	-0.18163	**-**0.84979	-0.86288	0.279555	0.042733	0.833508	0.911421	-0.16532
p-value	4.69E-01	0.08105	0.5008	3.08E-05	1.69E-05	0.2944	0.8751	6.05E-05	9.05E-07	0.5406

**Table 2:** Correlation coefficients between the right flanking length and composition difference (right ATP_flanking – non_ATP) for each amino acid

	**I**	**L**	**V**	**C**	**A**	**G**	**M**	**F**	**Y**	**W**
Correlation coefficient	-0.1635	0.791152	0.410819	0.531454	0.665265	-0.7964	0.163617	0.485453	-0.04779	0.510287
p-value	5.45E-01	2.62E-04	0.1139	0.03413	0.004918	0.000223	5.45E-01	5.66E-02	8.61E-01	4.34E-02
	H	K	R	E	Q	D	N	S	T	P
Correlation coefficient	-0.38939	0.074529	-0.2761	0.807045	0.746647	-0.47301	-0.60543	-0.89214	-0.92875	-0.19277
p-value	1.36E-01	7.84E-01	0.3006	0.000158	0.000891	0.06425	0.01295	3.41E-06	2.07E-07	0.4744

**Figure 3:**
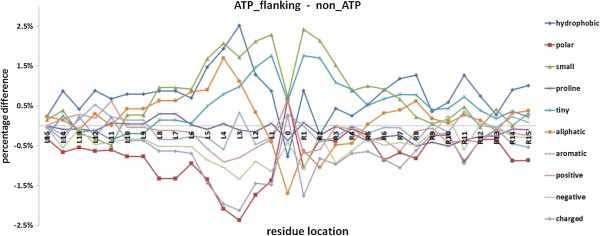
**Differences in physicochemical properties between AIRs and non-AIRs.** The values at position 0 on the horizontal axis mean the values for the AIRs, L means the left flanking side and R means the right flanking side.

Difference between the flanking regions and general non-AIRs regions with respect to 10 physicochemical properties is shown in Figure 3. The corresponding Pearson correlation coefficients between the flanking length and each physicochemical property difference are shown in Table 3 (for the left flanking side) and Table 4 (for the right flanking side). For the left flanking side, correlation coefficients related to small, tiny, negative and charged have absolute value > 0.60 at the *p*-value < 0.001; for the right flanking side, correlation coefficients related to small, tiny and aliphatic have absolute value > 0.73 at the *p*-value < 0.001. This phenomenon illustrates that, these physicochemical properties of flanking regions are very different from those of general non-AIRs regions.

**Table 3:** Correlation coefficients between the left flanking length and each physicochemical property difference (left ATP_flanking - non-ATP)

	**Hydrophobic**	**Polar**	**Small**	**Proline**	**Tiny**	**Aliphatic**	**Aromatic**	**Positive**	**Negative**	**Charged**
Correlation coefficient	0.20548	-0.35369	0.803793	-0.20618	0.802904	-0.07958	-0.30385	-0.44621	-0.72413	-0.60791
p-value	4.45E-01	1.79E-01	0.000176	0.4436	0.000181	7.70E-01	2.53E-01	8.32E-02	1.51E-03	1.25E-02

**Table 4:** Correlation coefficients between the right flanking length and each physicochemical property difference (right ATP_flanking - non-ATP)

	**Hydrophobic**	**Polar**	**Small**	**Proline**	**Tiny**	**Aliphatic**	**Aromatic**	**Positive**	**Negative**	**Charged**
Correlation coefficient	0.523796	-0.39506	-0.76834	-0.12863	-0.75009	0.732382	0.299015	-0.13031	0.570493	0.322113
p-value	3.73E-02	1.30E-01	0.000507	0.635	0.000818	1.25E-03	2.61E-01	6.31E-01	2.10E-02	2.24E-01

The above analyses of the composition and physicochemical properties for the three regions illustrated that flanking regions are highly relevant to the ATP-binding sites. Therefore, we assumed that the ATP-binding sites in protein sequences are highly contextual. Because ATP-binding sites are found to be more conserved than surrounding residues [7-10,13,18], we considered incorporating contextual information of residues with local evolutionary conservation to improve the prediction of ATP-binding sites.

### Prediction model

**Figure 4:**
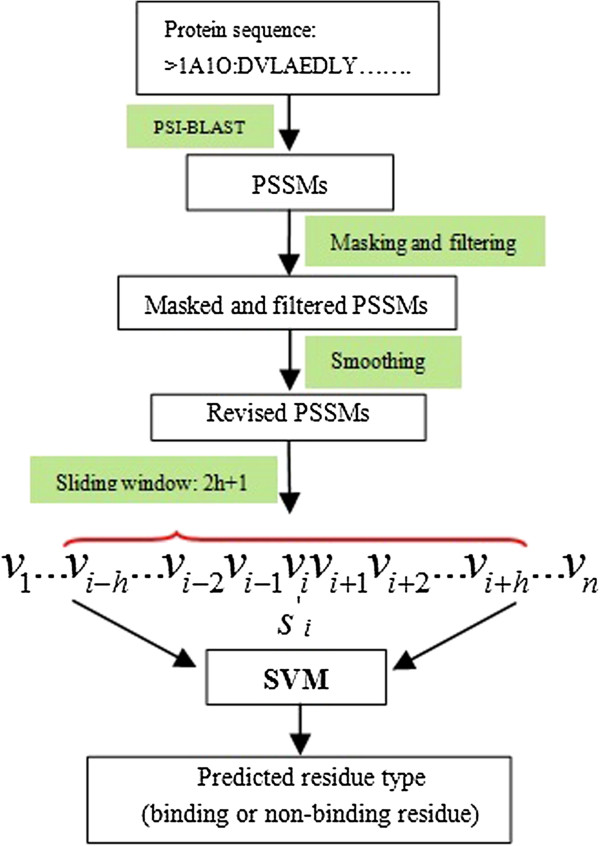
**Prediction model.** The length of sliding window is represented by *2 h+1*, n is the length of sequence, and *v*_*i*_ represents the corresponding amino acids *i* in the feature vector.

Our approach focuses on using high local evolutionary conservation scores in the PSSMs for prediction. The prediction model is shown in Figure 4.

### Evolutionary information (PSSM)

Evolutionary information was obtained from PSSMs generated by PSI-BLAST [19] with searching against NCBI non-redundant (nr) database [20] by three times iteration with an e-value of 0.001. Evolutionary information for each amino acid was encapsulated in a vector of 20 dimensions. The size of PSSM of a protein with N residues is 20 × N, where N is the length of a protein. 20 dimensions were considered as a standard amino acid size.

### Masking and filtering the PSSM

The modified PSSM is used to describe the local evolutionary information of each residue in a protein. It is converted from a standard PSSM according to formula (1) and (2).

Firstly, a masking sliding window with appropriate size is used to calculate the mean conservation score for each residue in a standard PSSM, and then, scores in PSSM are converted to local conservation scores according to formula (1). After that, the local conservation scores below the average scores are changed to 0 according to formula (2). This can strengthen the high conservative information while filtering out the low ones.

(1) $$\text{Masking}\_C_{i}=C_{i}-\frac{1}{2n+1}\sum_{i-n}^{i+n}C_{j}$$

(2) $$\mathrm{Filtering}\_C_{i}=\left\{\begin{array}{l} \mathrm{Masking}\_C_{i},\left(\mathrm{Masking}\_C_{i}>0\right)\\ 0,\left(\mathrm{Masking}\_C_{i}\leq 0\right) \end{array}\right)$$

**Figure 5:**
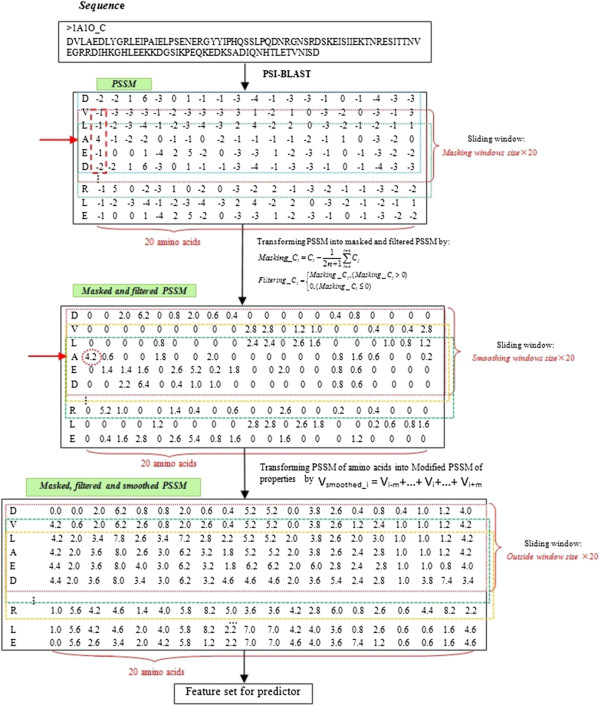
Procedure of preparing feature sets for the predictor.

*Masking*_*C*_*i*_ is the mean conservation score of residue *i*, *C*_*i*_ is the standard conservation score in PSSM, *2n+1* is the masking window size. Figure 5 illustrates an example of a masked and filtered PSSM.

### Smoothing the modified PSSM

Every value in a standard PSSM is calculated based on the assumption that the position of each value in the matrix is independent from the others. However, ligand-binding residues tend to appear continuously. The flanking regions also largely affect the binding behavior. In order to incorporate the dependency on surrounding neighbors of a central residue, we adopt the same smoothing method as Cheng-Wei C’s group [21], firstly, in order to deal with the N-terminal and C-terminal of a protein sequence, m (m is an odd number) ZERO vectors are appended to the head and the tail of a standard PSSM profile, where *2 m+1* is the size of a smoothing sliding-window. Then the smoothing sliding-window is used to incorporate the evolutionary information from upstream and downstream residues. Each row vector of an amino acid residue Ci is smoothed according to formula (3).

(3) $$\mathrm{Smoothing}\_C_{i}=\sum_{i-m}^{i+m}\mathrm{Filtering}\_C_{j}$$

Finally, each value in the smoothed PSSM matrix is scaled to the range of [-1, 1] according to a certain ratio. Procedure of preparing feature sets for the predictor is shown in Figure 5.

### Support vector machines (SVM) and 5-fold cross-validation

Identification of ATP-binding sites can be addressed as a two-classification problem, i.e., determining whether a given residue is binding residue or not. In this study, like the other researches, we adopted SVM to build the binary classifier. The prediction models were trained by the libSVM software package which was written by in Chih-Jen [22,23]. Here, the Radial Basis Function (RBF kernel) was adopted to construct the SVM classifiers. The grid search method [22,23] was used to search for the best parameters c and g for training. The negative samples were selected randomly with an equal number of positive samples. 5-fold cross-validation was used to evaluate the performance of the developed models, that is, the patterns were randomly divided into five sets. Four sets were used for training and the remaining one set was used for testing. The process was repeated until each set was used once for testing.

### Evaluation criteria

We adopted the evaluation criteria used in CASP10 [24]. The area under the corresponding ROC curve (AUC), MCC, and the accuracy (ACC) were adopted to evaluate the performance of the classifiers. The ROC plots with the AUC values were created by using the R statistical package [25]. The sensitivity, specificity, true positive rate (TPR), false negative rate (FPR) and ACC are defined as follows:

(4) $$\mathrm{Specifity}=\frac{\mathrm{TN}}{\mathrm{TN}+\mathrm{FP}}$$

(5) $$\mathrm{TPR}=\mathrm{Sensitivity}=\frac{\mathrm{TP}}{\mathrm{TP}+\mathrm{FN}}$$

(6) $$\mathrm{FPR}=1-\mathrm{Specificity}=\frac{\mathrm{FP}}{\mathrm{TN}+\mathrm{FP}}$$

(7) $$\mathrm{ACC}=\frac{1}{2}\left(\mathrm{Sensitivity}+\mathrm{Specificity}\right)$$

Where TP, TN, FP and FN represents true positive, true negative, false positive and false negative respectively.

## Results and discussion

### Window sizes

For developing the CLCLpred model, 3 window sizes are necessary; the masking-window size which is used to calculate average local conservation scores, the outside sliding-window size which would finally decide the dimensions of feature vectors, and the inside smoothing-window size which is used to strengthen the local conservation features. Here, for a fair comparison, we chose the same size 17 with the researches [10,16,17] as the outside-sliding window size. The masking-window size has the similar meaning with the outside sliding-window size. Both of them indicate the length of flanking regions that would be considered to affect a central residue. So, 17 was also adopted as our masking window size. With an outside sliding-window and a masking-window size of 17, the CLCLpred model was further tested according to different smoothing window sizes. The relative best ROC plots would be chosen to represent the performance of the related models.

### Effectiveness of the feature extracting methods

•Effectiveness at the individual protein level

To confirm the effectiveness of our feature extracting method in distinguishing AIRs from non-AIRs and to determine how this might benefit the predication, we selected the protein [PDB: 2B6F] as an example. It has a continuous ATP-binding region of “G-G-C-H-R” located at residues 81~ 85 in the A chain.

**Figure 6:**
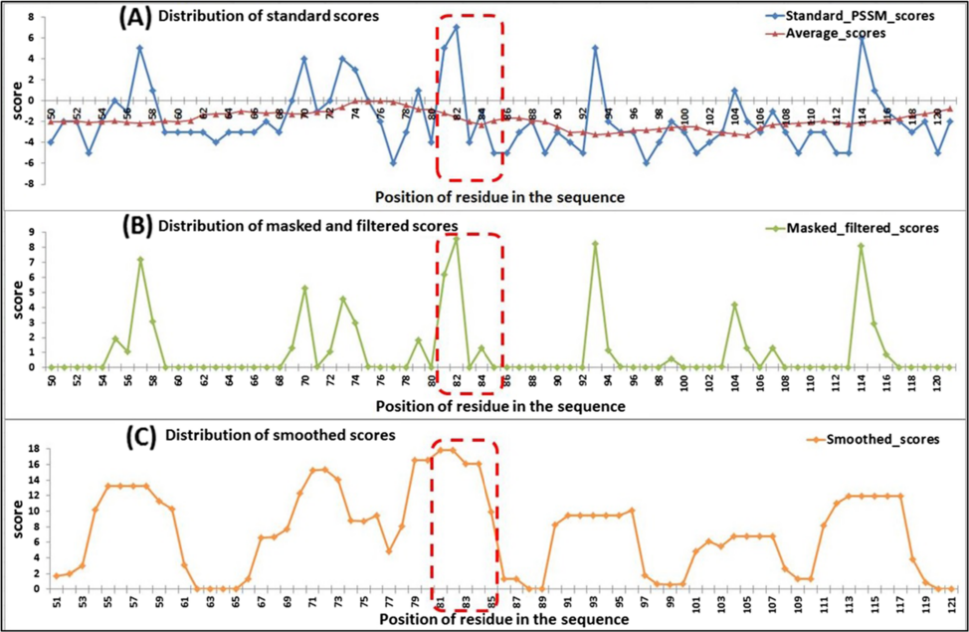
**Distribution of conservation scores for protein 2B6F (PDB ID) related to the three steps. (A)** distribution of scores in the standard PSSM, **(B)** distribution of scores in the masked and filtered PSSM, and **(C)** distribution of scores in the smoothed PSSM. The red dotted rectangles mark the position of ATP-binding residues (only residues 51~121 in the **A** chain are displayed).

Because the binding region “G-G-C-H-R” contains two residues of ‘G’, we extracted the ‘G’ column of position-specific scores from the standard PSSM (there are a total of 20 columns, corresponding to the 20 standard amino acids). Then, the distribution of the scores in the standard PSSM, masked PSSM, and smoothed PSSM were counted (Figure 6). In the standard PSSM, the distribution of scores between the AIRs and non-AIRs showed no distinct difference. The AIR region contains both highly conserved residues (‘G’) and highly variable residues (other amino acids). In the masked PSSM, scores below the average have been filtered out, thereby discarding the noise data (low conservative scores), that are undesirable for prediction. In the smoothed PSSM, scores of residues that are surrounded by conserved residues have been enhanced after smoothing, and scores of residues that are surrounded by poorly conserved residues have been weakened. Since AIRs are found to be more conserved than surrounding residues and flanked by less conserved residues [7-10,13,18], they are easy to appear as highly conserved peaks compared to the non-AIR regions. We have shown the results for just the ‘G’ column, similar results were obtained for ‘C’, ‘H’ and ‘R’ columns of the PSSM (data not shown).

•Effectiveness at the whole dataset level.

We next analyzed the distributions of local conservation scores for all residues in the data set ATP168 in three stages: in the standard PSSM, after masked and filtered, and after smoothed. We assumed that the summation of scores in each row in the corresponding PSSM represented the local conservation of the related residues *C*_*i*_. Then the local conservation score in each stage is able to be calculated according to formula (8~10).

**Figure 7:**
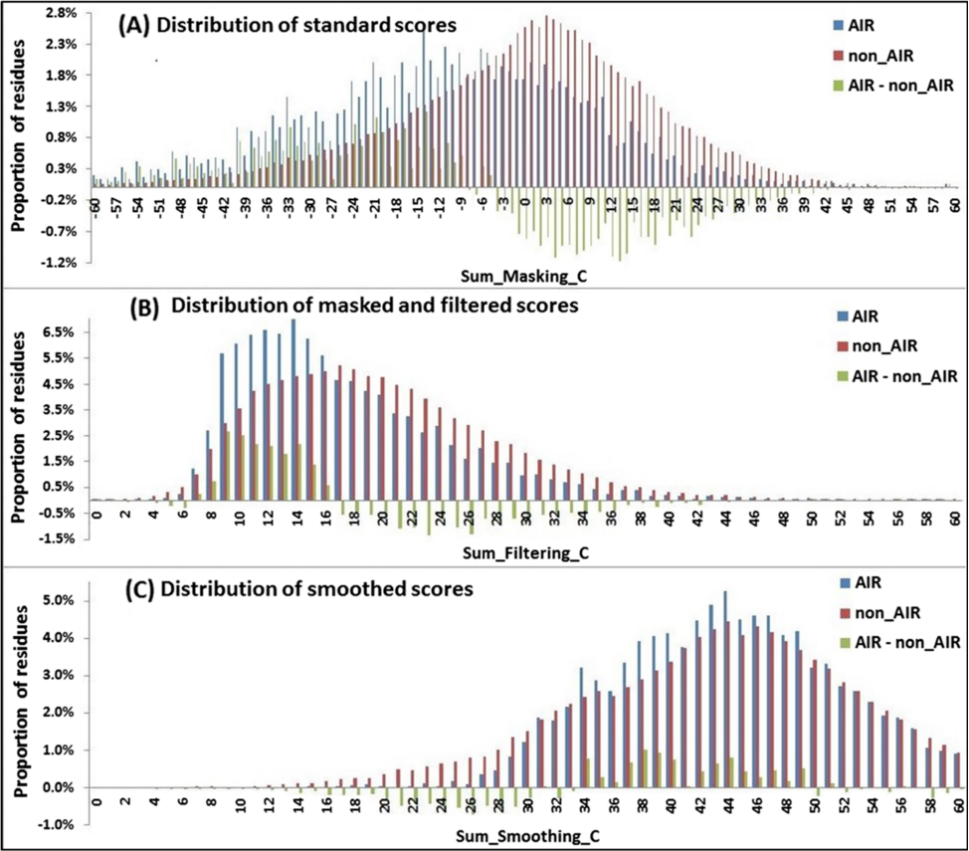
**Distribution of the summation scores of AIRs and non-AIRs related to the three steps. (A)** distribution of the summation scores in the standard PSSM **(B)** distribution of the summation scores in the masked and filtered PSSM, and **(C)** distribution of the summation scores in the smoothed PSSM.

Distribution of summation score related to The AIRs and non-AIRs was compared (Figure 7). In the standard PSSM, more non-AIRs showed stronger conservation than the AIRs. However, that, after filtered, the percentage of the AIRs with high local conservation scores increased significantly (Figure 7(B)). This can illustrate that, most of filtered out scores were belonging to the non-AIRs. Finally, after smoothed, more AIRs showed stronger local conservation than the non-AIRs (Figure 7(C)). Unsurprisingly, most of the AIRs and their flanking residues have stronger local conservation than the general non-AIRs. Because in order to maintain certain function, the functional sites of proteins must maintain a high degree of conservation. Figure 7 shows that, our feature extracting method can effectively extract the local conservation information of residues to distinguish the AIRs from non-AIRs.

### Performance of the CLCLpred and comparison with other PSSM-based methods

**Figure 8:**
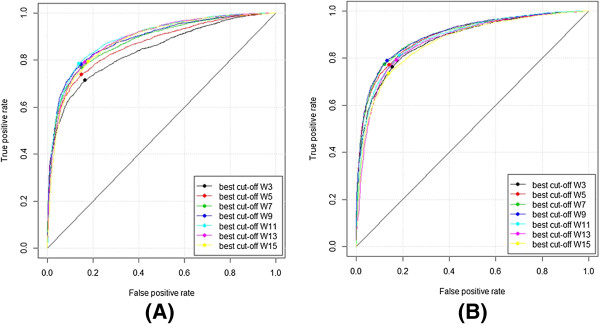
ROC plots of CLCLpred with different smoothing window sizes applied on ATP168 (A) and ATP227 (B) (W indicates the flanking window length).

**Figure 9:**
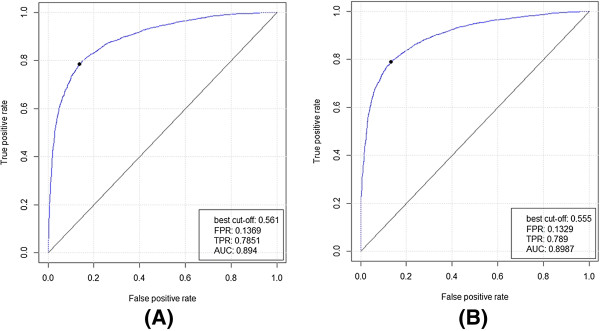
The best ROC plot of the CLCLpred method tested on ATP168 (A) and ATP227 (B).

ROC plots of the CLCLpred method apply to the ATP168 and ATP227 with different masking window sizes are shown in Figure 8. The respective best plots are shown in Figure 9.

### Performance comparison with other PSSM-based methods

**Figure 10:**
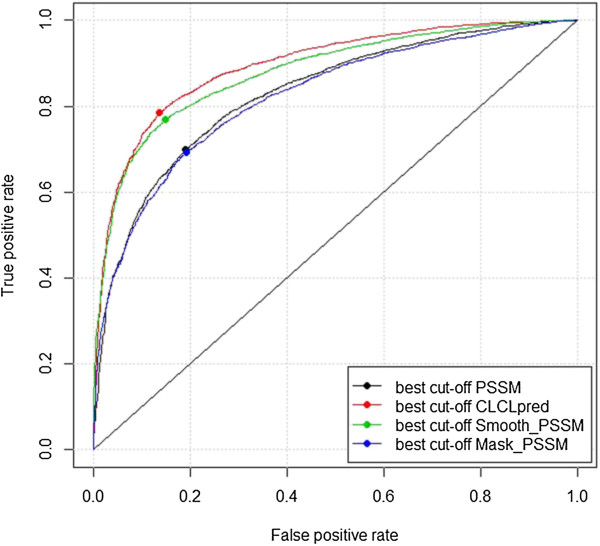
ROC plots of the four PSSM-based methods verified on ATP168.

In order to verify the importance of revising PSSM to match the detailed conservation pattern of ATP-binding sites, we have compared the CLCLpred method with three other PSSM-based methods on the ATP168 dataset: 1) the ‘PSSM’ method, which uses the direct output of PSSMs for prediction; 2) the ‘Smooth_PSSM’ method, which uses smoothed PSSMs without masking and filtering; and 3) the ‘Mask_PSSM’ method, which uses masked and filtered PSSMs without smoothing. Performances of the four methods are shown in Table 5, and the ROC plots of them are shown in Figure 10. The results demonstrate that, although all the four methods are based on PSSM of sequence, the CLCLpred method achieves the best performance.

**Table 5:** Performance comparison with other three PSSM-based methods on ATP168

**Dataset**	**Method**	**ACC**	**TPR**	**FPR**	**AUC**
ATP168	CLCLpred	0.824	0.785	0.137	0.894
	PSSM	0.755	0.700	0.191	0.823
	Smooth_PSSM	0.811	0.770	0.149	0.879
	Mask_PSSM	0.750	0.692	0.192	0.823

(8) $$\mathrm{Sum}\_\mathrm{Masking}\_C_{i}=\sum_{j=1}^{20}\mathrm{Masking}\_C_{j}$$

(9) $$\mathrm{Sum}\_\mathrm{Filtering}\_C_{i}=\sum_{j=1}^{20}\mathrm{Filtering}\_C_{j}$$

(10) $$\mathrm{Sum}\_\mathrm{Smoothing}\_C_{i}=\sum_{j=1}^{20}\mathrm{Smoothing}\_C_{j}$$

We speculate that the reasons for the above results include: 1) the direct outputs of PSSMs contain redundant features, because the ‘Mask_PSSM’ method which filters out the low local conservation scores achieves nearly the same performance with the ‘PSSM’ method; 2) ATP-binding sites in protein sequences are highly contextual, and incorporating the conservation information from sequentially neighboring residues results in improved performance. As can be seen from Figure 10, the methods with the smoothing step (‘Smooth_PSSM’ and CLCLpred) significantly outperform the methods without the smoothing step (‘Mask_PSSM’ and ‘PSSM’); and 3) although the ATP-binding sites are affected by neighboring residues, they maintain a certain degree of independence (Figure 1 also shows that 68% of binding-sites appear alone). This kind of functional site with their flanking regions usually contains both highly conserved residues and highly variable residues and is highly locally conserved on the whole. Without the masking and filtering steps, the predictive power of highly conserved residues will be weakened by noise data (low conservation scores from the neighboring residues) after smoothing. On the contrary, if the noise data are discarded by masking and filtering, the predictive power of intensively highly conserved residues will be strengthened after smoothing. Therefore, the CLCLpred method outperformed the ‘Smooth_PSSM’ method. The above results also confirmed the previous analysis [13] that, better exploiting the signal from sequentially neighboring residues would largely facilitate ligand-binding sites prediction.

### Performance comparison with other existing predictors

**Figure 11:**
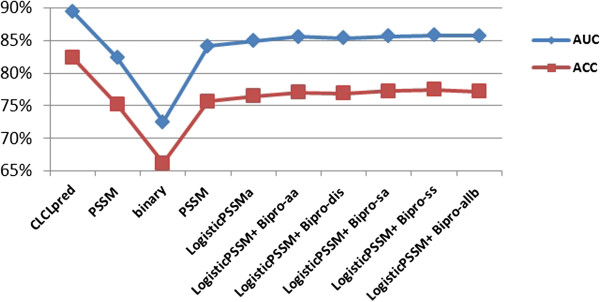
Detailed comparison of ACC and AUC for all the predictors on ATP168.

**Figure 12:**
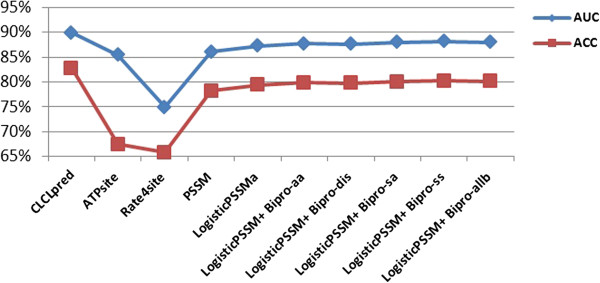
Detailed comparisons of ACC and AUC for all the predictors on ATP227.

Since we have extracted the data set ATP168 and ATP227 from the research papers [10,16,17], here, we also listed them out for the comparison. All the methods were trained and tested on the same data sets. Results are shown in Table 6. Detailed comparison of ACC and AUC among all the methods on ATP168 and ATP227 are shown in Figures 11 and 12 respectively. As shown in Table 6, Figures 11 and 12, the CLCLpred predictor achieved the best ACC and AUC.

**Table 6:** Performance of CLCLpred and the existing predictors tested on the ATP168 and ATP227

**Dataset**	**Method**	**ACC**	**TPR**	**FPR**	**AUC**
ATP168	CLCLpred (our proposed method)	0.824	0.785	0.137	0.894
	PSSM (reference [10])	0.752	0.700	0.196	0.823
	binary (reference [10])	0.663	0.655	0.330	0.725
	PSSM (reference [17])	0.757	0.757	0.243	0.841
	LogisticPSSMa (reference [17])	0.765	0.763	0.234	0.849
	LogisticPSSM+ Bipro-aa (reference [17])	0.770	0.769	0.228	0.855
	LogisticPSSM+ Bipro-dis (reference [17])	0.769	0.766	0.229	0.854
	LogisticPSSM+ Bipro-sa (reference [17])	0.772	0.770	0.225	0.856
	LogisticPSSM+ Bipro-ss (reference [17])	0.775	0.774	0.224	0.858
	LogisticPSSM+ Bipro-allb (reference [17])	0.772	0.770	0.227	0.857
ATP227	CLCLpred (our proposed method)	0.828	0.789	0.133	0.899
	ATPsite (reference [16])	0.675	0.361	0.012	0.854
	Rate4site (reference [16,26])	0.658	0.446	0.130	0.749
	PSSM (reference [17])	0.782	0.783	0.218	0.861
	LogisticPSSMa (reference [17])	0.794	0.792	0.204	0.873
	LogisticPSSM+ Bipro-aa (reference [17])	0.798	0.798	0.201	0.877
	LogisticPSSM+ Bipro-dis (reference [17])	0.798	0.797	0.201	0.876
	LogisticPSSM+ Bipro-sa (reference [17])	0.800	0.800	0.199	0.880
	LogisticPSSM+ Bipro-ss (reference [17])	0.802	0.801	0.197	0.881
	LogisticPSSM+ Bipro-allb (reference [17])	0.801	0.800	0.197	0.880

### Feature dimensions comparison

Previous researches [10,16,17] combined many predicted features with PSSM as their input for the ATP-binding prediction. These predicted features not only increased the complexity of the algorithm, but also increased the dimensions of feature vectors. We summarized them in Table 7 for a comparison. Table 7 demonstrates that, the vector dimension of CLCLpred is lower than those of the methods which incorporated other predicted features, such as predicted secondary structure (ss), predicted solvent accessibility (sa), predicted disorder probabilities (dis) and predicted dihedral angle. In machine leaning, if the number of training samples is limited, increasing the feature dimensions will be harmful for the classification.

**Table 7:** Summarization of feature types and number of vector dimensions

**Method**	**Feature types**	**Dimensionality**
CLCLpred	Modified PSSM	340
PSSM	PSSM	340
binary	Binary amino acid composition	340
ATPsite	PSSM+predicted dihedral angle+ss	NA
Rate4site	3D-structure	NA
LogisticPSSMa	LogisticPSSM	340
LogisticPSSM+ Bipro-aa	LogisticPSSM+amino acid composition	374
LogisticPSSM+ Bipro-dis	LogisticPSSM+predicted dis	374
LogisticPSSM+ Bipro-sa	LogisticPSSM+predicted sa	374
LogisticPSSM+ Bipro-ss	LogisticPSSM+predicted ss	374
LogisticPSSM+ Bipro-all	LogisticPSSM+predicted dis+sa+ss	510

## Conclusions

In this study, we proposed a simple method which adopted a modified PSSM encoding scheme for ATP-binding predication. In this approach, only the high local evolutionary conservation scores in PSSMs are considered as input, without employing any predicted features from other classifiers. By means of masking, filtering and smoothing, the modified PSSM combines predictive features which can distinguish the AIRs from non-AIRs effectively. When comparing with 10 other existing methods that used direct output of PSSMs or incorporated various predicted structural features as their input on the same datasets, our method achieved the best performance besides its minimum feature dimensions, i.e., achieving an ACC of 4.9%~16.1% and an AUC of 0.036~0.169 higher than other methods when tested on ATP168; achieving an ACC of 2.6%~17.0% and an AUC of 0.018~0.15 higher than other methods when tested on ATP227. These results demonstrate that, the local evolutionary conservation is distinct enough to enable an accurate prediction of ATP-binding sites directly from amino acid sequence, and incorporating other predicted features for prediction is not always helpful. A free Web server has been developed (http://webapp.yama.info.waseda.ac.jp/fang/LigandPred.php), which allows users to identify ATP-binding residues in a given sequence using the model trained on our data sets. Our CLCLpred model can be also used for identifying other ligand-binding sites of proteins.

## Abbreviations

ATP: Adenosine-5'-triphosphate; AIRs: ATP-interacting residues; non-AIRs: non-ATP interacting residues; PSSM: Position-specific scoring matrix; SVM: Support vector machine; ROC: Receiver operating characteristic; AUC: Area under the corresponding ROC curve; (PDB): Protein data bank; TPR: True positive rate; FPR: False negative rate.

## Competing interests

The authors declare that they have no competing interests.

## Authors’ contributions

CF carried out the implementation and drafted the manuscript, TN and HY read and revised the final manuscript, all authors read and approved the final manuscript.
